# Genotypes of SLC22A4 and SLC22A5 regulatory loci are predictive of the response of chronic myeloid leukemia patients to imatinib treatment

**DOI:** 10.1186/s13046-017-0523-3

**Published:** 2017-04-18

**Authors:** Monika Jaruskova, Nikola Curik, Rajna Hercog, Vaclava Polivkova, Eliska Motlova, Vladimir Benes, Hana Klamova, Pavla Pecherkova, Petra Belohlavkova, Filip Vrbacky, Katerina Machova Polakova

**Affiliations:** 1 Institute of Hematology and Blood Transfusion, U Nemocnice 1, 12820 Prague, Czech Republic; 2 Institute of Clinical and Experimental Hematology, 1st Medicine Faculty, Charles University, Prague, Czech Republic; 3 Institute of Pathophysiology, 1st Medicine Faculty, Charles University, Prague, Czech Republic; 4 European Molecular Biology Laboratory, Genomics Core Facility, Heidelberg, Germany; 5 Faculty of Science, Charles University, Prague, Czech Republic; 6 CELL, the Czech Leukemia Study Group for Life, Brno, Czech Republic; 7 4th Department of Internal Medicine Hematology, Charles University Faculty Hospital and Faculty of Medicine, Hradec Kralove, Czech Republic

**Keywords:** SLC, CML, Imatinib, Response

## Abstract

**Background:**

Through high-throughput next-generation sequencing of promoters of solute carrier and ATP-binding cassette genes, which encode drug transporters, we aimed to identify SNPs associated with the response to imatinib administered for first-line treatment of patients with chronic myeloid leukemia.

**Methods:**

*In silico* analysis using publicly available databases was done to select the SLC and ABC genes and their promoters for the next-generation sequencing. SNPs associated with the imatinib response were identified using Fisher’s exact probability tests and subjected to the linkage disequilibrium analyses with regulatory loci of concerned genes. We analyzed cumulative achievement of major molecular response and probability of event free survival in relation to identified SNP genotypes in 129 CML patients and performed multivariate analysis for determination of genotypes as independent predictors of outcome. Gene expression analysis of eight cell lines naturally carrying different genotypes was performed to outline an impact of genotypes on the gene expression.

**Results:**

We observed significant differences in the frequencies of the rs460089-GC and rs460089-GG (*SLC22A4*) genotypes among rs2631365-TC (*SLC22A5*) genotype carriers that were associated with optimal and non-optimal responses, respectively. Loci rs460089 and rs2631365 were in highly significant linkage disequilibrium with 12 regulatory loci in introns of *SLC22A4* and *SLC22A5* encoding imatinib transporters. Genotype association analysis with the response to imatinib indicated that rs460089-GC carriers had a significantly higher probability of achieving a stable major molecular response (*BCR-ABL1* transcript level below or equal to 0.1% in the international scale). In contrast, the rs460089-GG represented a risk factor for imatinib failure, which was significantly higher in rs460089-GG_rs2631365-TC carriers.

**Conclusions:**

This exploratory study depicted potentially important genetic markers predicting outcome of imatinib treatment, which may be helpful for tailoring therapy in clinical practice.

**Electronic supplementary material:**

The online version of this article (doi:10.1186/s13046-017-0523-3) contains supplementary material, which is available to authorized users.

## Background

Chronic myeloid leukemia (CML) is characterized by the Philadelphia chromosome (Ph) and fusion oncogene *BCR-ABL1* and is currently very treatable with tyrosine kinase inhibitors (TKIs) that inhibit the tyrosine kinase activity of the chimeric BCR-ABL1 protein. Imatinib is still prevalently used in clinical practice as the first-line treatment for newly diagnosed CML patients. However, resistance to imatinib occurs in approximately 20-30% of CML patients [1–3]. Known mechanisms of resistance to TKIs include the overexpression of the *BCR-ABL1*, additional cytogenetic abnormalities and mutations in the kinase domain of BCR-ABL1 [4]. In addition, the reduced bioavailability of imatinib in leukemic cells is an important pharmacokinetic factor that contributes to resistance development.

The activity and expression of drug transporters affect the intracellular concentration of a drug. Solute carrier (SLC) proteins facilitate drug entry into cells, whereas ATP binding cassette (ABC) transporters facilitate drug excretion. The human genome contains 49 ABC transporter-encoding genes, of which 10 have been associated with multidrug resistance of cancer cells [5]. The best-studied multidrug efflux transporter in CML is ABCB1, which is overexpressed in blast crisis and contributes to the reduced effectiveness of chemotherapy in advanced disease [6, 7].

The large group of 55 SLC gene families encodes 362 transporters. Seven SLC gene families encode transporters functionally annotated as drug carriers [8]. Major molecular response (MMR, i.e., ≤0.1% BCR-ABL1^IS^; IS – International Scale) is frequently observed during imatinib treatment regardless of the daily dose in patients with increased SLC22A1 (OCT1) activity. Patients with normal or reduced activity of this transporter benefit from an increase in the imatinib dose and achieve MMR more frequently than patients who received standard doses [9, 10].

Single nucleotide polymorphisms (SNPs) in the coding regions of several imatinib carrier genes have been studied in connection with the treatment response. For example, SNP rs683369 in exon 2 of *SLC22A1* has been associated with poor response to imatinib in CML patients of Asian origin [11]. Angelini et al. [12] recently reported that the *SLC22A4* rs1050152-C allele was significantly associated with MMR in the studied CML patients. Associations of the *MDR1* rs2032582, rs1128503 and rs60023214 genotypes with the imatinib response have also been reported, although the results were quite discordant [13–15].

Transporter expression can be significantly affected by polymorphisms in the promoters of transporter-encoding genes, and these polymorphisms may have an important impact on drug distribution and, consequently, on the response. More than 500 polymorphisms have been reported in 107 promoters of the ABC and SLC gene families in healthy people from different ethnic groups [16].

Here, we present an exploratory work studying an association between SNPs identified using high-throughput next-generation sequencing (NGS) screening in the promoters of genes encoding drug carriers and the imatinib response in CML patients.

## Methods

### Patients and samples

The set-up of the patient cohort was critical for initial high-throughput SNP screening in the promoter regions of 19 genes with the aim to reveal genotypes associated with imatinib response. Therefore, we selected patients treated in daily clinical practice of hematological center followed strict criteria to making the maximum effort to eliminate potential biases in the analyses. The criteria included 1) imatinib first line treatment; 2) good patient compliance; and 3) standardized and validated molecular and clinical data. We paid attention to the inclusion of comparable sizes of cohorts of patients optimally responding to imatinib first-line treatment and non-optimally responding patients, to statistically evaluate differences in SNP allele frequencies between the cohorts.

Eighty three CML patients treated in the Institute of Hematology and Blood Transfusion, Prague (UHKT) were selected for initial SNP screening (Additional file 1: Table S1). The imatinib responses were classified according to the European LeukemiaNet (ELN) recommendations [3] and the patients were scored at a landmark of 12 months as optimal responders (*BCR-ABL*1 ≤ 0.1% IS; *n* = 40) or non-optimal responders (*BCR-ABL1* > 0.1% IS and/or Ph+ > 0%; *n* = 43; failure and warning) (Additional file 1: Table S1). During the study, it was possible to enlarge the cohort of CML patients de novo-diagnosed at the UHKT and of CML patients treated at the University Hospital Hradec Kralove, Czech Republic, due to our collaboration. In total, 46 patients with a minimal follow-up time of 12 months since the initiation of imatinib treatment were added following the inclusion criteria listed above (Additional file 1: Table S1).

Total leukocytes from the peripheral blood of patients were used for DNA and RNA isolation.

This work was conducted in accordance with the principles of the Declaration of Helsinki and was approved by the Ethics Committees of the UHKT Prague and University Hospital Hradec Kralove. All patients provided written informed consent for the use of their samples for this research work.

### Primer design and PCR of promoter regions

The sequences covering the promoters of the 19 genes were analyzed using The National Center for Biotechnology Information (NCBI), Pharmacogenetics of Membrane Transporters Database (UCSF PMT) and The Eukaryotic Promoter Database (EPD). We designed primers to generate amplicons with a length of 1000–1500 bp encompassing the whole promoter region, including the proximal parts, and occasionally first exons and introns using Primer3 plus [17], Primer-BLAST [18] and OligoAnalyzer 3.1 [19] (Additional file 2: Table S2). Genomic DNA was isolated from patients in the testing cohort using TRIzol® (Thermo Fisher Scientific, Waltham, MA USA) or guanidinium thiocyanate (SERVA Electrophoresis GmbH, Heidelberg, Germany) from bio-banked cell lysates with a concentration of 10x10^6^ cells/ml. To generate the amplicon library for NGS analysis, PCR was performed using the designed primer pairs (Additional file 2: Table S2) and the FastStart™ High Fidelity PCR System (Roche Applied Science, Basel, Switzerland) or AccuPrime Supermix I (Thermo Fisher Scientific) Taq polymerases following 3 PCR protocols (Additional file 3: Table S3). The PCR products were verified by electrophoresis in an agarose gel and imaging under a UV transilluminator and quantified in the 2100 Bioanalyzer (Agilent Technologies, Santa Clara, CA USA).

### Amplicon next-generation sequencing analysis

Primers for SNP genotyping were designed for 15 SLC and 4 ABC genes encoding transporters functionally annotated as drug carriers through the plasmatic membrane (Additional file 2: Table S2). PCR conditions are summarized in Supplement (Additional file 3: Table S3).

Amplicons from 38 patients were sequenced using the GS Junior system (454 technology; Roche Applied Science). Amplicon libraries were prepared using the Rapid Library Preparation kit following the manufacturer’s manual (Roche Applied Science). Nebulization of PCR products to the required approximate lengths of 400–600 bp was performed under 55 psi of nitrogen pressure for 2.5 min. Fragment end repair, AMPure bead preparation, adaptor ligation, and small fragment removal were then performed according to the manufacturer’s manual (Roche Applied Science). The quality of the prepared amplicon libraries was determined with the 2100 Bioanalyzer (Agilent Technologies) using the High Sensitivity DNA chip (Agilent Technologies). The KAPA Library Quantification Kit for the Roche 454 GS Titanium platform (Kapa Biosystems, Wilmington, MA USA) was applied for nebulized amplicon library quantitation. In each NGS run, 19 amplicons from 12 patients were analyzed. Thus, 12 commercially available Multiple Identifiers (MIDs; Roche Applied Science) were used in the adaptor ligation step to distinguish the sequence data. Nineteen amplicons from 12 patients were pooled in an equimolar ratio, and the pooled samples were processed by emulsion PCR following the emPCR Amplification Manual Lig-L (Roche Applied Science). Sequencing was performed according to the Sequencing Manual using appropriate chemistry (Roche Applied Science). Due to the availability of a new kit (GS Junior + Sequencing Kit XL+; Roche Applied Science) for long amplicon sequencing, the amplicon fragmentation step was omitted in the amplicon analyses of 24/38 patients.

Amplicon libraries of the 19 amplicons in another 45 patients were sequenced using MiSeq (Illumina, San Diego, CA USA).

Sequence analysis and SNP identification were performed using NextGENe software (Softgenetics, State College, PA USA).

### Linkage disequilibrium analysis

Linkage disequilibrium (LD) analysis of genotyped SNPs and proxies identified in the *SLC22A4* and *SLC22A5* non-coding regions was performed using the LDlink 1.1 database [20]. LDmatrix, which is an interactive heatmap matrix of the pairwise LD statistics, was performed on the European population. SNPs with significant RegulomeDB scores 1–3 for known DNA regulatory elements were explored using the LDproxy utility. The haplotype frequencies were evaluated using the LDhap utility.

### Calculation of the *BCR-ABL1* halving time

The *BCR-ABL1* transcript level data were obtained from regular monitoring every 3 months during imatinib treatment. *BCR-ABL1* mRNA quantification is standardized in our laboratories (Prague and Hradec Kralove) within the EUTOS for CML project of ELN, and data are reported in the International Scale (IS) [21, 22]. In 2008, laboratories standardized their *BCR-ABL1* RT-qPCR techniques. Prague achieved a conversion factor from EUTOS reference laboratory in Mannheim. Hradec Kralove was referred to Prague. Therefore, in all patients, who were diagnosed from 2004 to 2007 (*n* = 35/129) the *BCR-ABL1* transcript levels were reanalyzed retrospectively with standardized *BCR-ABL1* RT-qPCR employing conversion factors. The halving time for the *BCR-ABL1/GUSB* transcripts (the rate of change of *BCR-ABL1* from each patient’s baseline value assessed during imatinib treatment by estimating the number of days required for *BCR-ABL1* to achieve one-half of the baseline value) was calculated according to the method of Branford et al. [23]. The *BCR-ABL1* halving time was calculated at a median of 6.2 months (4.5–8.9) of imatinib treatment for the enlarged patient cohort.

### Genotyping of SNPs in the *SLC22A4* and *SLC22A5* promoters

SNPs in the *SLC22A4* and of *SLC22A5* promoters were analyzed in the group of patients added (*n* = 46) to the cohort during the work on this study and in nine tested cell lines using Sanger sequencing. Amplification was performed using the FastStart™ High Fidelity PCR System (Roche Applied Science). The PCR conditions are summarized in additional file (Additional file 3: Table S3c). The PCR products were purified using the QIAquick PCR Purification Kit (Qiagen, Hilden, Germany). Sequencing PCR was performed using the BigDye® Terminator v3.1 Cycle Sequencing Kit (Thermo Fisher Scientific). Briefly, 1.5 μl of purified PCR product was added to a reaction mixture containing 1 μl of 5× Sequencing Buffer, 1 μl of Big Dye v3.1, and 10 pmol of the forward or reverse primer. The cycling conditions were as follows: 25 cycles of 96°C for 10 s, 55°C for 10 s, and 60°C for 4 min. The sequencing PCR products were purified using a DyeEx v2.0 Spin kit (Qiagen). Purified samples were dried in a SpeedVac SPD 111V P1 (Thermo Fisher Scientific), resolved in 25 μl of formamide (Thermo Fisher Scientific) and denatured. Sequencing of both strands was performed on the ABI PRISM 3500 (Thermo Fisher Scientific). Mutation Surveyor software v3.10 (Softgenetics) was used for sequence analyses and SNP scoring.

### Genotyping of SNP rs1050152

The TaqMan® SNP Genotyping Assay was used to genotype SNP rs1050152 (Thermo Fisher Scientific). The StepOnePlusTM system and appropriate software (Thermo Fisher Scientific) were used to perform the analysis and SNP scoring.

### Cell lines

Established leukemia and lymphoma cell lines KCL-22 (CML; DSMZ no. ACC 519), K562 (CML; DSMZ no. ACC 10), SUP-B15 (BCR-ABL1+ B cell precursor leukemia; DSMZ no. ACC 389), Z-138 (Mantle cell lymphoma; ATCC no. CRL-3001), CML-T1 (CML; DSMZ no. ACC 7), JURKAT (T cell leukemia; DSMZ no. ACC 282), MAVER-1 (B cell lymphoma; DSMZ no. ACC 717), RAMOS (Burkitt lymphoma; DSMZ no. ACC 603) were obtained from publicly accessible biological resource centers (Leibniz Institute–Deutsche Sammlung von Mikroorganismen und Zellkulturen GmbH/DSMZ, Braunschweig, Germany; ATCC, Manassas, VA, USA). No cell lines used in this work are listed in the database of commonly misidentified cell lines. Cells were authenticated by providers and tested for Mycoplasma contamination using MycoAlert PLUS detection kit (LT07-705; Lonza Group AG, Basel, Switzerland). The latest testing for Mycoplasma contamination was up to 1 month after cells thawing. Cell lines were handled and cultivated in appropriate medium according the recommendations of the supplier. The cells were used in experiment within 2 months after thawing.

### Expression analysis of *SLC22A4* and *SLC22A5* in cell lines

Total RNA was isolated from cell lysates stored in TRIzol® (Thermo Fisher Scientific) at a concentration of 1x10^6^ cells/ml. The RNA quantity was determined on the NanoDrop ND-1000 Spectrophotometer (Thermo Fisher Scientific). A 200 ng of RNA was transcribed to cDNA using iScript cDNA Synthesis Kit (#170-8891; BioRad Laboratories, Hercules, CA, USA). TaqMan® Gene Expression Assays Hs01548718_m1 for *SLC22A4* and Hs00929869_m1 for *SLC22A5* (Thermo Fisher Scientific) were used for relative quantification on 7900HT Fast Real-time PCR System (Thermo Fisher Scientific). Glyceraldehyde 3-phosphate dehydrogenase (*GAPDH*) was used as the housekeeping gene. The delta Ct calculation was used to evaluate relative quantity.

### Statistical analysis

For allele frequency analysis in the patient cohorts, Fisher’s exact probability test (2x2 or 2x3) was applied using online utilities [24]. The associations between genotypes and the *BCR-ABL1* halving time were analyzed using the Kruskal-Wallis test. Cumulative incidence curves of stable MMR achievement during imatinib treatment were calculated using the Mann-Whitney test. The Kaplan-Meier method was employed to estimate event-free survival (EFS). Events were defined as loss of response, *BCR-ABL1* mutation, progression or death related to CML during imatinib treatment. The censored patients also included those whose treatment was switched to 2nd line TKIs. Graphical analysis was supplemented using the log-rank test. Univariate and multivariate analyses were performed to identify whether the SNP alleles can predict outcome. Multivariate analyzes was performed by logistic regression (MMR) and the Cox proportional hazard regression (EFS). Statistical analyses were conducted using MATLAB version R2015b.

## Results

### *In silico* analysis of the SLC and ABC genes

Analysis using the NCBI, UCSF PMT and EPD databases demonstrated that the SLC and ABC gene groups are highly diverse and that their individual genes are scattered throughout the genome. Promoters differed greatly in the length of the promoter sequences and/or in the distance from the transcription start site (Additional file 4: Table S4). For SNP screening in the promoter regions by NGS, we selected 19 transporter-encoding genes with annotated drug transportation functions that were expressed in the liver (because the liver greatly contributes to imatinib metabolism), small intestine (imatinib absorption), bone marrow and leukocytes (targets of imatinib in CML) (Additional file 2: Table S2).

### Identification of SNPs in the promoters of SLC and ABC genes associated with patient response

**Fig. 1:**
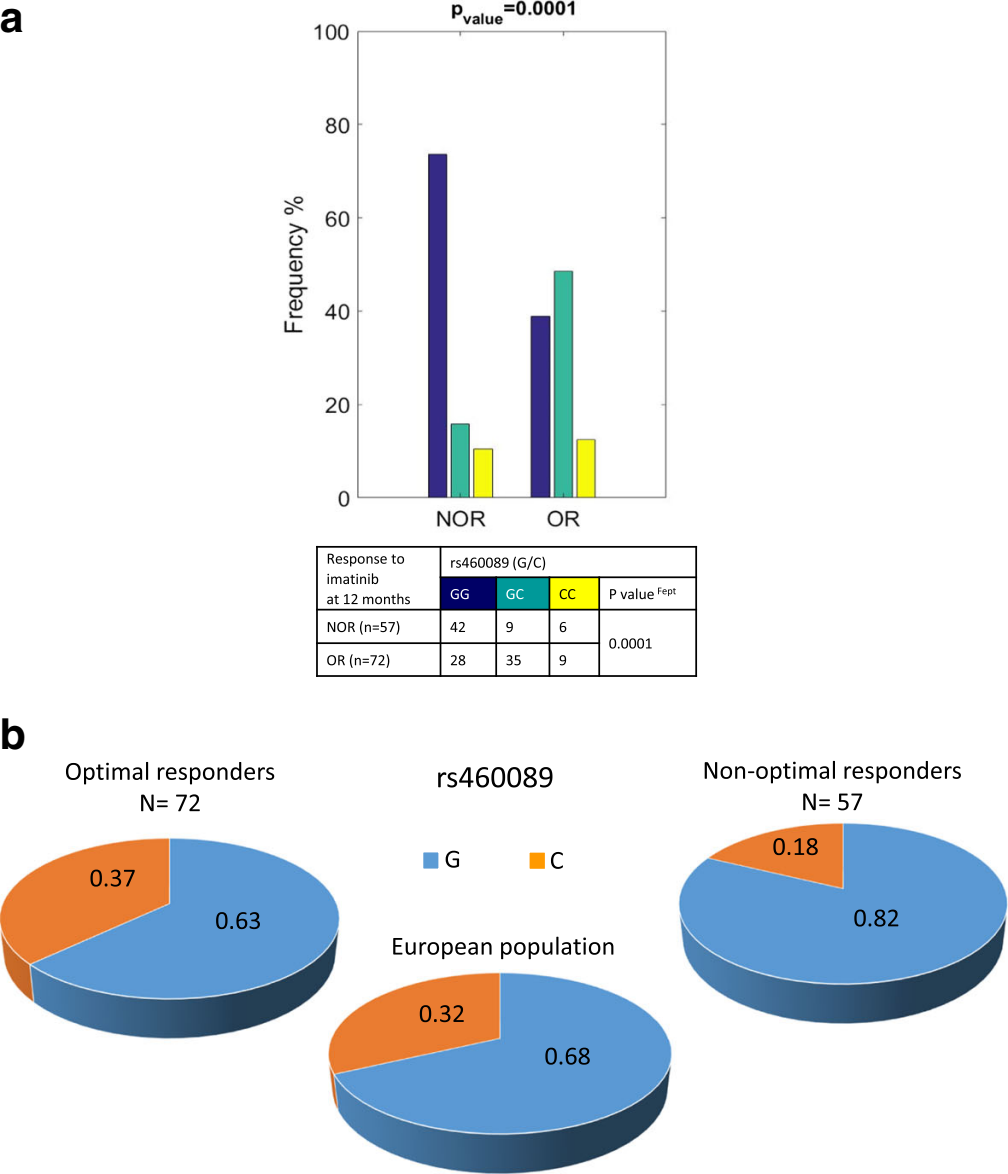
**a** Genotype frequencies of the rs460089 and rs460271 in patients with optimal and non-optimal response to imatinib at 12 months. Note – the graphs illustrate the frequencies of the genotypes of rs460089, which exactly reflect the genotype frequencies of rs460271; NOR–patients with non-optimal response to treatment; OR–patients with optimal treatment response. **b** Differences in the frequencies of the G and C alleles of rs460089 in European CML patients with non-optimal responses to imatinib compared to the European population

Initial SNP screening of the 19 promoters was performed in 83 CML patients who fulfilled the criteria (see Methods, section Patients and samples, and Additional file 1: Table S1). We detected 95 SNPs within the 1486 amplicons analyzed. The primer design allowed identification of 12 SNPs outside the promoter regions; altogether 9 SNPs in exon 1 of genes *SLC22A1*, *SLC22A5*, *SLC22A8*, *SLC01A2* and 3 SNPs in intron 1 of genes *SLC28A1*, *SLC28A3*, *SLC47A2* (Additional file 4: Table S4). Based on Fisher’s exact probability tests, 2 SNPs in high LD were identified in the promoter of *SLC22A4* (rs460089 G/C, no. 29, and rs460271 G/C, no. 30; Additional file 5: Figure S1) to be associated with imatinib response. Frequency of GG genotypes of both SNPs were significantly higher in patients non-optimally responding to imatinib at 12 months, whereas GC genotypes were significantly associated with an optimal response (*P* = 0.003; Additional file 5: Figure S2). SNP genotyping for the promoters of *SLC22A4* and *SLC22A5* were performed in the group of patients (*n* = 46; Additional file 1: Table S1) who were added to the cohort during the study. The same frequency distribution of the rs460089 and rs460271 (*SLC22A4*) genotypes was observed, although this result was not significant due to the low number of patients (Additional file 5: Figure S2). In the enlarged cohort of CML patients (*n* = 129), a higher frequency of GG in non-optimal responders and a higher frequency of GC in optimal responders were observed, with a markedly increased level of significance (*P* = 0.0001; Fig. 1a). The G and C allele frequencies among the non-optimal responders highly differed from the frequencies in the European population (Fig. 1b).

The Fisher’s exact probability test analysis revealed other potentially interesting SNPs in LD located in the *SLC22A5* promoter including the 5′ UTR showing more frequent heterozygous genotype in non-optimal responders compared to optimal responders (no. 35, 36, 37, 38 and 39, Additional file 5: Figure S1). However, the differences of the genotype frequencies of these 5 SNPs in the enlarged cohort of CML patients (*n* = 129) were not significant (Additional file 5: Figure S3a).

Based on observation of Angelini et al. [12] we performed genotyping analysis of rs1050152 located in exon 9 of *SLC22A4*. A slightly higher frequency of the rs1050152-CT was observed in patients optimally responding to imatinib, but this finding was not significant (Additional file 5: Figure S3b).

### Linkage disequilibrium analysis

LD analysis of the genotyped SNPs in the *SLC22A4* and *SLC22A5* was performed using the LDlink 1.1 database [20]. The SNPs rs60978556, rs369724970 and rs202088921 *(SLC22A5)* were excluded from the analysis because the minor alleles were detected in a maximum of only four cases (Additional file 5: Figure S1, no. 40–42). The genotyped SNP rs1050152 previously reported to be associated with the imatinib response [12] was included in the analysis.

**Fig. 2:**
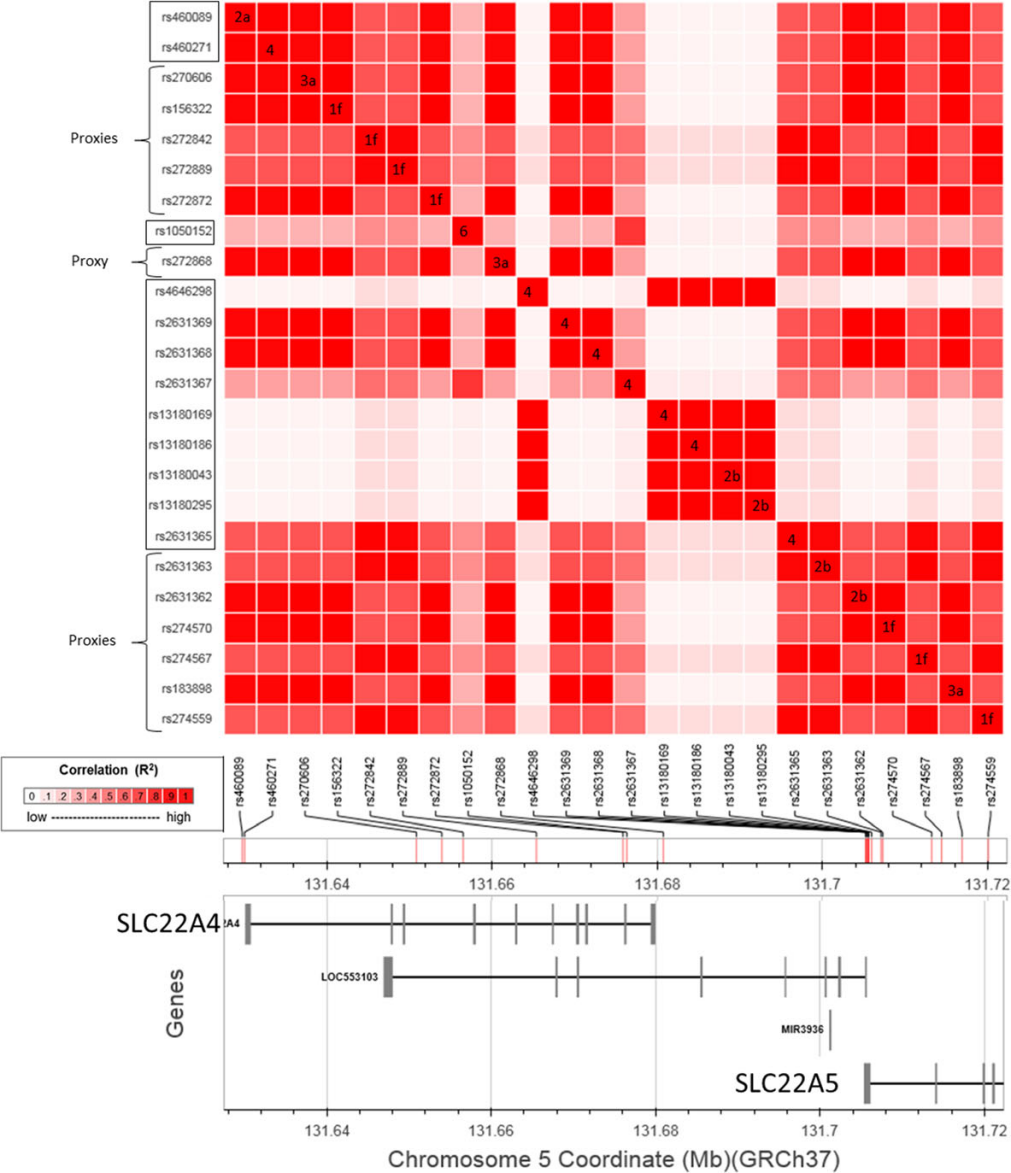
Linkage disequilibrium matrix of genotyped SNPs and their proxies. SNPs in boxes were genotyped. SNPs with RegulomeDB scores for known DNA regulatory elements were explored using the LDproxy utility. The numbers in each SNP coordinate indicate the RegulomeDB score, which reflects the degree of likelihood of affecting the binding of transcription regulatory proteins, DNA motifs, and chromatin structure

Using the LDmatrix utility, we created an interactive heatmap matrix of pairwise LD statistics for the genotyped SNPs and their identified proxies in the European population (Fig. 2). We observed two groups of genotyped SNPs that were in significant pairwise LD with proxy SNPs with annotated regulatory functions (R^2^ = 0.98–1.0; *P* < 0.0001). The first group consisted of the loci rs460089 and rs460271 (*SLC22A4*), rs2631369 and rs2631368 (*SLC22A5*), which were in LD with the proxy SNPs (Fig. 2; R^2^ = 0.98–1.0; *P* < 0.0001) with annotated regulatory functions rs270606, rs156322, rs27287 and rs272868 (*SLC22A4*), rs2631362 and rs274570 (*SLC22A5*) and rs183898 (*SLC22A5*) (Table 1). Moreover, the RegulomeDB [25] database indicated that the genotyped locus rs460089 likely affects transcription factor binding (Table 1b). The reference sequence of this locus is a conserved sequence for the DNA binding proteins identified in the blast crisis CML cell line K562. According to the Position-Weight Matrix (PWM) for transcription factor binding, this locus is a conserved site for the MYC:MAX heterodimer.

**Table 1:** Regulatory likelihood of the investigated SNPs according to the RegulomeDB database

a.							
Gene Functional consequence	SNP	Position	Regulome DB score	eQTL (Monocytes)	Histone modification Chromatin state Blood cells	Protein binding K562	
*SLC22A4* Intron 3	rs156322 A/G	chr5:131653924	1f	*SLC22A4* *SLC22A5*	*Quiescent/Low* T, B and NK cells from PB *Strong transcription* Primary neutrophils, monocytes, MNC from PB *Weak transcription* Primary HSC *Genic enhancers* K562	NA	
*SLC22A4* Intron 3	rs272842 C/T	chr5:131656516	1f	*SLC22A4* *SLC22A5*	*Quiescent/Low* T, B and NK cells from PB *Strong transcription* Primary neutrophils and monocytes from PB *Weak transcription* Primary HSC, MNC from PB, K562	NA	
*SLC22A4* Intron 5	rs272889 C/T	chr5:131665377	1f	*SLC22A4* *SLC22A5*	*Quiescent/Low* Primary T, B and NK cells from PB *Strong transcription* MNC from PB *Weak transcription* T-helper naïve from PB, K562, primary HSC	NA	
*SLC22A4* Intron 8	rs272872 C/T	chr5:131675863	1f	*SLC22A4* *SLC22A5*	*Quiescent/Low* primary T, B and NK cells from PB *Strong transcription* MNC from PB *Weak transcription* T-helper naïve from PB, K562, primary HSC	NA	
*SLC22A5* Intron 1	rs2631363 C/T	chr5:131707094	2b	NA	NA	RBBP5, POLR3A	
*SLC22A5* Intron 1	rs2631362 C/T	chr5:131707292	1f	*SLC22A4* *SLC22A5*	*Enhancers* MNC and T cells from PB *Flanking active TSS* K562 *Weak transcription* MNC from PB, primary HSC	RBBP5	
*SLC22A5* Intron 1	rs274570 A/G	chr5:131713225	1f	*SLC22A4* *SLC22A5*	*Quiescent/Low* Primary T cells from PB *Weak transcription* primary HSC, MNC from PB, K562	NA	
*SLC22A5* Intron 2	rs274567 A/G	chr5:131714408	1f	*SLC22A4* *SLC22A5*	*Quiescent/Low* T regulatory and helper cells, primary neutrophils from PB *Strong transcription* K562, primary T cells from PB, primary HSC G-CSF mobilized female and male *Weak transcription* primary HSC, primary T, B and NK cells from PB	NA	
*SLC22A5* Intron 2	rs183898 C/G	chr5:131716901	3a	NA	*Quiescent/Low* Primary neutrophils from PB, T regulatory and helper cells from PB *Weak Transcription* B cells, MNC form PB, K562, primary HSC short term culture	NA	
*SLC22A5* Intron 3	rs274559 C/T	chr5:131720069	1f	*SLC22A4* *SLC22A5*	*Quiescent/Low* primary neutrophils from PB, T regulatory and helper cells from PB *Strong Transcription* K562 *Weak Transcription* primary HSC, primary NK cells from PB, T and B cells from PB, MNC from PB	NA	
b.							
Gene Functional consequence	SNP	Position	Regulome DB score	Histone modification Chromatin state Blood cells	Protein binding K562	Motif (cell type)	Position-Weight Matrix for TF binding Ref. sequence of SNP
*SLC22A4* Promoter	rs460089 C/G	chr5:131629771	2a	NA	POLR2A, PHF8, HMGN3, UBTF, CTCF, ATF3, USF1, MAX, MYC, CBX3, UBTF, ZBTB7A, CCNT2, E2F6, IRF1, BCLAF1, ETS1	MYC:MAX (K562)	
*SLC22A4* Intron 3	rs270606 C/T	chr5:131650866	3a	NA	HDAC8	STAT5A (NA)	
Downstream from *SLC22A4* (LOC553103 intron 3)	rs272868 C/G	chr5:131680750	3a	*Quiescent/Low* Primary T and B cells from PB *Strong transcription* MNC from PB *Weak transcription* T-helper naïve from PB, K562, primary HSC	STAT1, STAT2, CEBPB	MEOX2 (NA)	
*SLC22A5* NC transcript variant, UTR variant 5 prime	rs13180043 C/T	chr5:131705586	2b	NA	POLR2A, PHF8, TAF1, ETS1, IRF1, MYC, UBTF, RBBP5, SAP30, MAX, E2F6, NRF1, HMGN3, KDM5B, E2F1	HIC1 (K562)	
*SLC22A5* NC transcript variant, UTR variant 5 primes	rs13180295 G/A	chr5:131705587	2b	NA	POLR2A, PHF8, TAF1, ETS1, IRF1, MYC, UBTF, RBBP5, SAP30, MAX, E2F6, NRF1, HMGN3, KDM5B, E2F1	HIC1 (K562)	

The table is divided into two sections for clarity. a. Summary of the eQTL, chromatin state in blood cells and protein binding in K562. b. Summary of protein binding in K562, motifs, Position-Weight Matrix (PWM) for transcription factor binding and chromatin state in blood cells. Legend: 1f–likely to affect binding and linked to expression of a gene target (eQTL + TF binding/DNase peak); 2a–likely to affect binding (TF binding + matched TF motif + matched DNase Footprint + DNase peak); 2b–likely to affect binding (TF binding + any motif + DNase Footprint + DNase peak); 3a–less likely to affect binding (TF binding + any motif + DNase peak)

The second group consisted of rs2631365 (*SLC22A5*), which was in LD with the proxy SNPs (Fig. 2; R^2^ = 0.98–1.0; *P* < 0.0001) rs272842 and rs272889 (*SLC22A4*), rs2631363, rs274567 and rs274559 (*SLC22A5*), which have annotated regulatory functions (Table 1).

Another group of SNPs that were identified in the RegulomeDB as likely affecting TF binding included genotyped rs13180043 and its adjoining SNP rs13180295 (LD; R^2^ = 1.0; *P* < 0.0001); both of these SNPs are located in the *SLC22A5* non-coding region. Transcription factor HIC1 bound the reference sequence of both SNPs, which was identified in K562 according to the PWM (Table 1b).

The genotyped SNP rs1050152 located in exon 9 of *SLC22A4* had minimal binding evidence according to the RegulomeDB and was in LD with SNPs rs2631367 (R^2^ = 0.79; *P* < 0.0001) and rs2631365 (R^2^ = 0.44; *P* < 0.0001) in *SLC22A5*. However, minimal binding evidence was assigned for both SNPs.

Based on this analysis, we next focused on the genotypes of rs460089 (*SLC22A4*), rs2631365 (*SLC22A5*), which were in LD with proxy SNPs, and rs13180043 with regulatory function (*SLC22A5*).

### The rs460089-GC is associated with a shorter *BCR-ABL1* halving time and better outcome

Next, we tested whether there were differences between the rs460089, rs2631365 or rs13180043 genotypes in relation to the early molecular response (EMR), which is defined as a *BCR-ABL1* transcript level of ≤10% at 3 months [3]. Data for EMR scoring were available for 117/129 patients. We did not find a significant link between the genotypes and the achievement of EMR (Table 2a). We are aware that EMR is defined based on a single measurement of the *BCR-ABL1/GUSB* transcript level, and variability in the single measurement might influence EMR scoring. Therefore, we decided to calculate the *BCR-ABL1* halving time (HT) at 6 months, when *BCR-ABL1/GUSB* transcript levels were available from three consecutive measurements in 119/129 (at the time of imatinib initiation, at 3 months and at 6 months). We found that the rs460089-GC was associated with a significantly shorter *BCR-ABL1* halving time compared with that of the rs460089-GG (*P* < 0.0001) (Table 2b). Significant differences between genotypes and the *BCR-ABL1* halving time were not found for rs2631365 and rs13180043 (Table 2b).

**Table 2:** Frequencies of genotypes of rs460089, rs2631365 and rs13180043, association of genotypes with EMR and BCR-ABL1 HT6 and frequencies of haplotypes in the European population

a.												
Response to imatinib at 3 months	rs460089 (G/C)				rs2631365 (T/C)				rs13180043 (C/T)			
	GG	GC	CC	*P* value ^Fept^	TT	TC	CC	*P* value ^Fept^	CC	CT	TT	*P* value ^Fept^
EMR (*n* = 86)	42	33	11	0.470	31	45	10	0.671	77	9	0	0.220
non-EMR (*n* = 31)	19	8	4		12	14	5		25	6	0	
b.												
	rs460089 (G/C)^a^				rs2631365 (T/C)^a^				rs13180043 (C/T) ^a^			
	GG	GC	median/*P* value^MMT^		TT	TC	median/*P* value^MMT^		CC	CT	median/*P* value ^MMT^	
HT6 median of days (number of cases)	33.10 (61)	21.25 (43)	27.00 p <0.00001		32.29 (44)	25.41 (54)	27.04 *p* = 0.223		26.46 (100)	28.34 (17)	27.00 *p* = 0.177	
c.												
	rs2631365-TC				rs13180043-CC							
	Optimal resp.	Non-optimal resp.	*P* value ^Fept^		Optimal resp.	Non-optimal resp.	*P* value ^Fept^					
rs460089-GG	6	15	0.00006		26	33	0.0023					
rs460089-GC	27	8			30	8						
rs460089-CC	4	0			9	6						
d.												
	rs460089-GG_ rs2631365-TC (number)	rs460089-GC_ rs2631365_TC (number)	median value/*P* value ^MMT^		rs460089-GG_ rs13180043-CC (number)	rs460089-GC_ rs13180043-CC (number)	median value/*P* value ^MMT^					
HT6 median of days (number of cases)	36.34 (18)	21.48 (34)	25.41/0.004		33.150 (50)	21.251 (37)	27.100/0.002					
e.												
Haplotype rs460089_rs270606_rs156322_rs272872_rs272868_rs2631362_rs274570_rs183898	European population											
G_G_T_G_G_A_C_C	0.68											
C_A_C_A_C_G_T_G	0.32											
G_A_C_A_C_G_T_G	0.003											
C_G_T_G_G_A_C_C	0.001											
Haplotype rs2631365_rs272842_rs272889_rs2631363_rs274567_rs274559												
T_G_G_A_C_A	0.58											
C_A_A_G_T_G	0.42											
C_A_G_A_C_G	0.004											
C_A_A_A_C_A	0.001											
C_G_G_A_C_A	0.001											

a. Differences in genotype frequencies of rs460089, rs2631365 and rs13180043 between patients with EMR and non-EMR to imatinib. b. The associations between genotypes and *BCR-ABL1* halving time at 6 months. c. Differences between the rs460089 genotype frequency in patients with an optimal response and non-optimal response to imatinib at 12 months in rs2631365-TC carriers and in rs13180043-CC carriers. d. Differences in the *BCR-ABL1* halving time between the genotypes clusters. e. Haplotypes frequencies in the European population. *Fept* Fischer exact probability test, ^*MMT*^ Mood’s median test

^a^Homozygous status of minor alleles was not considered due to low number of cases

**Fig. 3:**
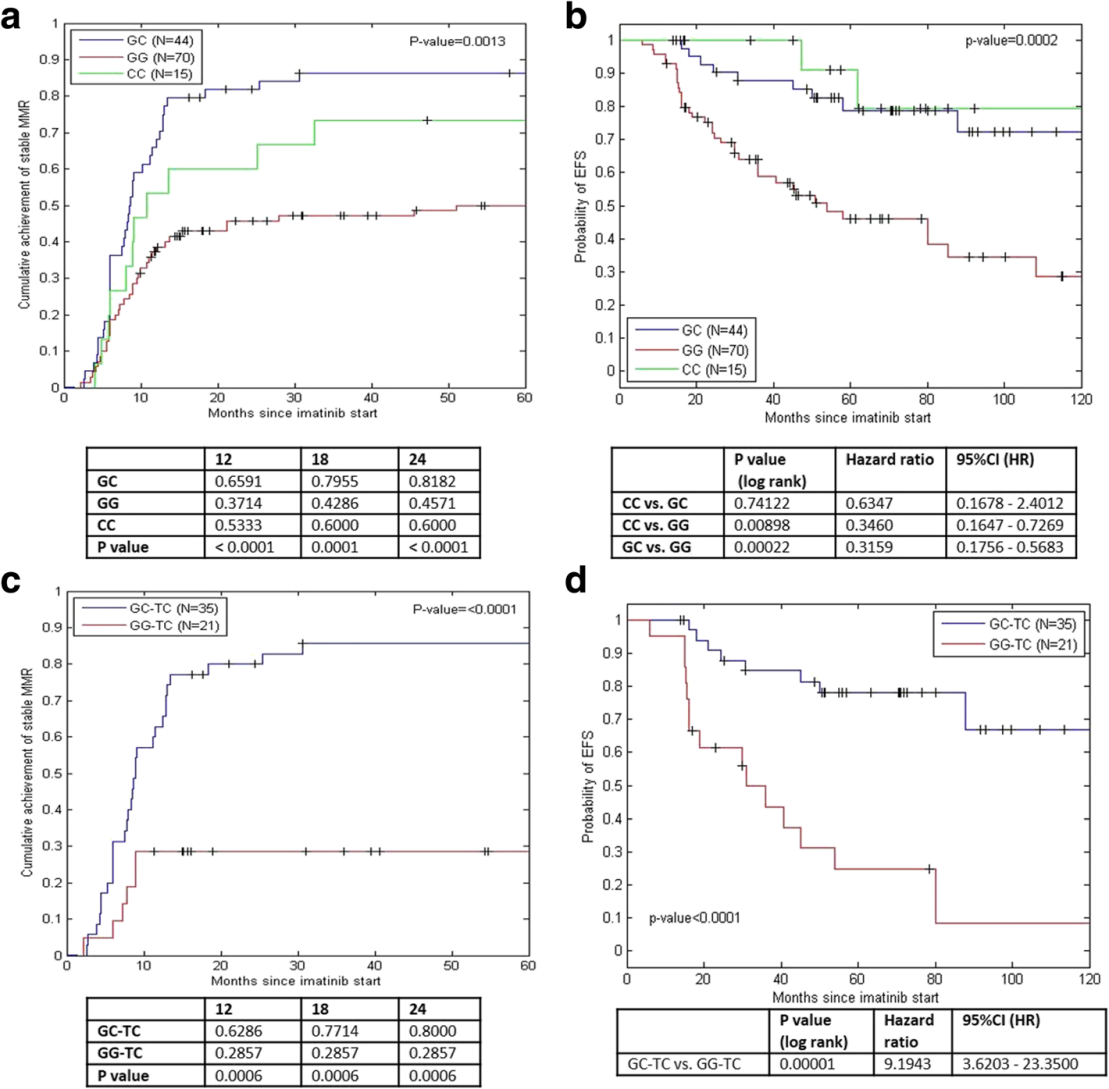
Cumulative achievement of stable MMR and EFS during imatinib treatment in association with genotypes. **a** CCI of stable MMR in rs460089-GC, rs460089-CC and rs460089-GG genotypes; the data in the table indicate the probability of achieving MMR at 12, 18 and at 24 months of imatinib therapy for a particular genotype. **b** EFS in rs460089-GC, rs460089-CC and rs460089-GG genotypes. **c** CCI of stable MMR in rs460089-GC_rs2631365-TC vs rs460089-GG_rs2631365-TC genotypes. **d** EFS in rs460089-GC_rs2631365-TC vs rs460089-GG_rs2631365-TC genotypes. Events were defined as loss of response, *BCR-ABL1* mutation, progression or death related to CML during imatinib treatment. The censored patients also included those whose treatment was switched to 2nd line TKIs. Graphical analysis was supplemented using the log-rank test

Importantly, we found that the rs460089-GC was significantly associated with cumulative achievement of stable MMR during imatinib treatment, in contrast to rs460089-GG (Fig. 3a). Consequently, patients with rs460089-GC had a significantly higher probability of EFS during imatinib treatment than patients with rs460089-GG (Fig. 3b). The rs460089-CC genotype showed similar trend with rs460089-GC in response and EFS, however due to the low number of cases, it is difficult to reliably interpret data.

### Identification of a cluster of genotypes associated with response and event-free survival

Using the Fisher’s exact probability tests we also determined that the frequencies of the rs460089-GG and rs460089-GC and their association with response differed significantly among rs2631365-TC carriers (60/129) (Table 2c). The rs460089-GG_rs2631365-TC genotype was more frequent in non-optimal responders, while the rs460089-GC_rs2631365-TC was significantly more frequent in optimally responding patients (*P* = 0.00006). Consequently, the rs460089-GG_rs2631365-TC was associated with a significantly longer *BCR-ABL1* HT6 compared with that of rs460089-GC_rs2631365-TC (*P* = 0.004; Table 2d). The rs460089-GC_rs2631365-TC was associated with the cumulative achievement of stable MMR during imatinib treatment compared with rs460089-GG_rs2631365-TC (Fig. 3c; *P* < 0.0001). Similarly, patients with the rs460089-GC_rs2631365-TC genotype exhibited a higher probability of survival without events compared with patients with the rs460089-GG_rs2631365-TC (Fig. 3d; *P* < 0.0001). The rs460089-GC_rs2631365-TC genotype likely reflects the heterozygous status of altogether 13 *SLC22A4* and *SLC22A5* regulatory loci based on data from the LD and LDhap analyses. Thus, the alleles of rs460089 and rs2631365 perfectly predicted the alleles of the proxy regulatory SNPs (Fig. 2; Table 2e).

As next, Fisher’s exact probability tests revealed that the frequency of rs460089-GC is significantly higher in optimally responding patients to imatinib at 12 months carrying rs13180043-CC genotype compared to non-responding patients with the same genotype cluster (Table 2c). *BCR-ABL1* HT6 was significantly shorter in patients with rs460089-GC_rs13180043-CC compared to patients with rs460089-GG_rs13180043-CC. It is important to note that 35/38 of rs460089-GC_rs13180043-CC carriers carried also rs2631365-TC genotype, while only 10/56 patients with rs460089-GG_rs13180043-CC carried also rs2631365-TC genotype.

### Multivariate analysis determined rs460089 predictive of outcome

We determined whether genotypes of rs460089 are predictive of outcome independently of other variables using multivariate analysis. In the multivariate models for MMR achievement and EFS, covariates with *P* < 0.05 were included (Table 3). Rs460089-GC was predictive of MMR achievement at any time points during imatinib treatment compared to rs460089-GG (odds ratio [OR] for rs460089-GC was 0.28, 95% confidence interval [CI] = 0.06–0.42; *P* = 0.0003). Rs460089-GC was predictive of superior EFS compared to rs460089-GG (for rs460089-GC the hazard ratio [HR] was 0.28, 95% CI = 0.14–0.59, *P* = 0.0008). The result of the analysis regarding to rs460089-CC genotype is difficult to reliably interpret due to a very low number of cases.

**Table 3:** Univariate and multivariate analysis of parameters predictive of outcome

	No MMR N (%)	MMR N (%)	OR	95% CI OR	*P* value	Number	Events N	HR	CI (95% HR)	*P* value
Univariate										
Imatinib dose										
Imatinib 400/day	33 (73)	75 (89)	0	0	0	108	37	0	0	0
Imatinib 200–300 mg/day	12 (27)	9 (11)	2.86	(1.16–7.89)	0.0251	21	11	1.64	(0.84–3.23)	0.1482
Risk score										
Eutos Low	31 (72)	74 (88)	0	0	0	105	34	0	0	0
Eutos High	12 (27)	10 (12)	2.86	(1.12–7.32)	0.0452	22	12	1.53	(1.10–2.13)	0.0121
Sokal Low	13 (31)	42 (50)	0	0	0	55	17	0	0	0
Sokal Intermediate	11 (26)	30 (36)	1.18	(0.47–3.00)	0.8130	41	9	0.79	(0.46–2.13)	0.5772
Sokal High	18 (43)	12 (14)	4.85	(1.86–12.65)	0.0019	30	19	1.69	(1.22–2.36)	0.0016
Hasford Low	11 (26)	30 (36)	0	0	0	41	11	0	0	0
Hasford Intermediate	18 (43)	47 (56)	1.04	(0.43–2.52)	1.0000	65	21	1.13	(0.55–2.35)	0.7344
Hasford High	13 (31)	7 (8)	5.06	(1.60–15.99)	0.0058	20	13	1.71	(1.14–2.55)	0.0095
SNPs										
rs460089										
GG	35 (78)	35 (42)	0	0	0	70	37	0	0	0
CC	4 (9)	11 (13)	0.36	(0.10–1.25)	0.1530	15	2	0.43	(0.21–0.87)	0.0197
GC	6 (13)	38 (45)	0.15	(0.06–0.43)	<0.0001	44	9	0.28	(0.13–0.58)	0.0006
rs13180043										
CC	35 (78)	77 (92)	0	0	0	112	41	0	0	0
CT	10 (2)	7 (8)	3.14	(1.11–8.94)	0.0321	17	10	1.91	(0.95–3.81)	0.0677
rs2631365										
TT	19 (42)	31 (37)	0	0	0	50	20	0	0	0
CC	6 (14)	13 (15)	0.75	0.24–2.32	0.7808	19	4	0.61	(0.35–1.05)	0.0767
TC	20 (44)	40 (48)	0.82	0.73–1.79	0.6903	60	24	1.00	(0.55–1.82)	0.9900
Multivariate										
Imatinib dose										
Imatinib 400/day	33 (73)	75 (89)	0	0	0	108	37	0	0	0
Imatinib 200–300 mg/day	12 (27)	9 (11)	4.17	(1.42–12.18)	0.0191	21	11	1.98	(1.01–3.90)	0.0481
SNP										
rs460089										
GG	35 (78)	35 (42)	0	0	0	70	37	0	0	0
CC	4 (9)	11 (13)	0.53	(0.27–1.02)	0.0740	15	2	0.40	(0.20–0.83)	0.0141
GC	6 (13)	38 (45)	0.28	(0.06–0.42)	0.0003	44	9	0.28	(0.14–0.59)	0.0008

Variables with *P* ≤ 0.05 in the univariate analysis were entered into a multivariate model and odds ratio and hazard ratio was computed for both type of analysis. Multivariate analyzes was performed by logistic regression (MMR) and the Cox proportional hazard regression (EFS). *P* value <0.05 was considered significant. Eutos score was not available in two patients, Hasford and Sokal scores were not available in three patients

### Gene expression in relation to genotypes

Based on the obtained data, we also investigated differences in the expression of *SLC22A4* and *SLC22A5* between the carriers of different genotypes. The chromatin state of the regulatory loci results in different *SLC22A4* and *SLC22A5* transcription levels among blood cell types (Table 1). Only lysates of patients’ total leukocytes from peripheral blood were available for this study. Leukocytes are a mixture of diverse types of mature cells; therefore, we decided to perform the experiment using cell lines (*n* = 8). In all cell lines tested, we found that the expression of *SLC22A4* was significantly lower (*P* = 0.02) than *SLC22A5*. All four tested cell lines with rs460089-GG and cell lines CML-T1 and MAVER-1 with rs460089-GC expressed *SLC22A5* levels that were lower compared with the levels in the JURKAT and RAMOS carrying rs460089-GC (Additional file 5: Figure S4a). Among the rs460089-GG carriers, only KCL-22 and SUP-B15 were rs2631365-TC carriers showing lower expression of *SLC22A5* compared with that of the rs460089-GC_rs2631365-TC carriers RAMOS, JURKAT and MAVER-1 (Additional file 5: Figure S4b).

## Discussion

Due to the development of second- and third-generation TKIs targeting BCR-ABL1, therapy for CML patients may be managed in a more personalized manner than ever before. Patients in whom specific *BCR-ABL1* kinase domain mutations develop during TKI treatment should be switched to another TKI that is sufficiently potent to overcome the *BCR-ABL1* mutation [2]. Clinical studies of newly diagnosed CML patients treated with nilotinib and dasatinib reported earlier achievement and higher rates of molecular response compared with imatinib [26]. An important portion of CML patients may significantly profit from the selection of second- generation TKIs as first-line [3]. In addition to cost considerations, the higher risk of toxicity is an important reason why physicians in daily clinical practice often do not consider second-generation TKIs as first-line treatment if the patient’s Sokal score is intermediate or low. Because kinase domain BCR-ABL1 mutations most likely do not exist at the time of diagnosis of chronic phase [2], there is a lack of molecular markers for predicting patient outcome at the time of diagnosis and facilitating the selection of an appropriate TKI for effective treatment. Pharmacogenetics represents a potential source of molecular markers because patients with a haplotype (an inherited set of DNA variations) associated with the key genes encoding drug transporters have genetic predispositions to respond optimally or non-optimally to drugs that are dependent on active transport through the plasmatic membrane. Studies of this issue have yielded discordant data [27]. The limitations include the use of heterogeneous patient cohorts and limited, pre-selected numbers of polymorphisms in the exons of the investigated genes [11, 12].

In this work, we identified genotypes that occurred with different frequencies in patients showing an optimal or non-optimal response to imatinib at 12 months after NGS screening of the promoters of 19 genes encoding drug transporters. The frequencies of the rs460089 genotypes (promoter of *SLC22A4*) differed significantly in association with the imatinib response. The rs460089-GC was significantly associated with the cumulative achievement of stable MMR during imatinib treatment and a higher probability of survival without events. A similar trend for rs460089-CC genotype was occurred, outlining also a superior response to imatinib, however due to a low number of cases (CC genotype is rarely occurred in the population), it is difficult to reliably interpret data.

We demonstrated that the genotyped rs460089 (*SLC22A4*), rs460271 (*SLC22A4*), rs2631369 (*SLC22A5*) and rs2631368 (*SLC22A5*) were in LD with six regulatory loci located in the introns of *SLC22A4* (*n* = 3/6) and *SLC22A5* (*n* = 3/6) as well as one locus located downstream of *SLC22A4*. Four loci have been demonstrated to be eQTLs in monocytes that regulate *SLC22A4* and *SLC22A5* expression. The 2 loci and the rs460089 allele are conserved regions for binding of transcription factors. Because the rs460089 locus is in highly significant LD with these 7 loci, the rs460089 alleles perfectly predict the alleles of the loci. Thus, we assume that the rs460089-GG is associated with unfavorable outcome due to transcriptional deregulation of *SLC22A4* and *SLC22A5* in this group of patients. For the genotyped rs2631365 (*SLC22A5*), we identified five regulatory loci in highly significant LD, including two in the introns of *SLC22A4* and three in the introns of *SLC22A5*.

The rs460089-GC_rs2631365-TC genotype was significantly more frequent in optimal responders to imatinib, corresponding to the significantly higher ratio of patients with this genotype who achieved stable MMR during imatinib therapy and exhibited a higher probability of EFS compared with patients with the rs460089-GG_rs2631365-TC. We assume that the rs460089-GC_rs2631365-TC reflects the heterozygous status of the 13 regulatory loci of *SLC22A4* and *SLC22A5* that are favorable for the optimal response to imatinib. In the heterozygous genotype, an allele on one chromosome and the corresponding allele on the homologous chromosome containing the two different copies of the allele might interact with one another to cause transvection [28, 29], which can lead to SLC22A4/SLC22A5 activation or repression. Data from the expression analyses in the tested cell lines indicated strong transcription of *SLC22A5* and very weak transcription of *SLC22A4*, independent of genotype. The higher expression of *SLC22A5* observed in rs460089-GC_rs2631365-TC genotype-carrying cell lines compared with rs460089-GG_rs2631365_TC carriers outlined the suggested impact of the genotype on the regulation of *SLC22A5* transcription and, consequently, on the intracellular concentration of imatinib. A large functional study is warranted to uncover a principle of suggested mutual regulation of alleles of loci of both genes on their expressions. Table 1 summarizes the regulatory regions, which we identified being in highly significant linkage disequilibrium with rs460089 and rs2631365 outlining the complexity of the *SLC22A4* and *SLC22A5* gene expression regulation. Binding or depletion of transcription factors in relation to certain combination of genotypes, analysis of histone modifications and DNA methylation levels of CpG islands in promoters should be studied in relation to SLC22A4 and SLC22A5 expression and to imatinib uptake in vitro.

Angelini et al. [12] previously reported that rs1050152-CT allele carriers had a significantly higher MMR compared with TT carriers during imatinib treatment. In this study, we observed a higher frequency of the rs1050152-CT in patients optimally responding to imatinib, although this difference was not significant. According to the RegulomeDB database, SNP rs1050152 is not regulative. Even when, rs1050152 SNP is not causative for the expression regulation of both genes, alleles are in linkage disequilibrium with alleles of regulatory loci. The significance of LD is not so high for rs1051052 to perfectly predict alleles of regulatory loci, however in the large cohort of CML patients the reported significantly higher MMR response rate to imatinib in rs1050152-CT carriers observed by Angelini et al. [12] may be found mirroring the causative genotypes associated with the superior response.

## Conclusions

In this exploratory study, we identified SNPs in regulatory regions of *SLC22A4* and *SLC22A5* genes, coding imatinib transporters, which were significantly associated with outcome of imatinib in CML patients. Multivariate analysis revealed that rs460089, a locus affecting transcription factor binding in the *SLC22A4* promoter, is an independent genetic parameter predicting outcome of first line imatinib therapy. Patients with the rs460089-GC would likely respond optimally of imatinib treatment. In contrast, the rs460089-GG represents a risk factor for the occurrence of events during imatinib therapy, which is significantly increased in rs460089-GG_rs2631365-TC carriers. This risk factor may be associated with a sub-lethal concentration of imatinib, which may lead to resistant clone development and disease progression. Observations of this exploratory work outlined that rs460089 and rs2631365 represent genetic markers for prediction of the response to imatinib and for tailored therapy management. This warrants a verification on a large cohort of CML patients before their introduction into a clinical practice.

## Additional files

Additional file 1: Table S1. Characteristics of the patients. (DOCX 28 kb)

Additional file 2: Table S2. The sequences of designed primers for amplification of selected promoters of SLC and ABC genes including annotations of transporters. (XLSX 11 kb)

Additional file 3: Table S3. PCR cycling conditions for the amplification of the promoter regions. (XLSX 11 kb)

Additional file 4: Table S4. Identified SNPs in amplified region including whole promoters of 19 selected genes in the cohort of 83 CML patients. (XLSX 20 kb)

Additional file 5: Figure S1. A colormap of genotypes distribution among optimally and non-optimally responding patients to first-line imatinib treatment at 12 months. Each square illustrates each genotyped SNP for each patient. *Red squares* = minor allele homozygotes; *pink squares* = heterozygotes; *white squares* = major allele homozygotes; *gray square* = not analyzed. **Figure S2.** Genotype frequencies of the rs460089 and rs460271 in patients with optimal and non-optimal response to imatinib at 12 months. 1 – Initial cohort of 83 patients; 2 – An independent group of added patients. Note – the graphs illustrate frequencies of genotypes of rs460089, which exactly reflect genotypes frequencies of rs460271. **Figure S3.** Genotype frequencies of a. rs13180043 (*SLC22A5*) and b. rs1050152 (*SLC22A4*, exon 9) in patients with optimal and non-optimal response to imatinib at 12 months. Note – the graph a. illustrates frequencies of genotypes of rs13180043, which exactly reflect genotypes frequencies of rs4646298, rs13180169, rs1310186, and rs13180295. **Figure S4.** Relative mRNA levels of *SLC22A4* and *SLC22A5* in tested cell lines. a. Graph shows expression in all eight cell lines. b. Graph shows expression of cell lines carrying rs460089-GG_rs2631365-TC or rs460089-GC_rs2631365-TC genotypes. (DOCX 687 kb)

## Abbreviations

ABC: ATP binding cassette
CML: Chronic myeloid leukemia
EFS: Event-free survival
ELN: European LeukemiaNet
EMR: Early molecular response
EPD: The Eukaryotic Promoter Database
HR: Hazard ratio
HT: Halving time
IS: International Scale
LD: Linkage disequilibrium
MMR: Major molecular response
NCBI: The National Center for Biotechnology Information
NGS: Next-generation sequencing
OR: Odds ratio
Ph: Philadelphia chromosome
PWM: Position-Weight Matrix
SLC: Solute carriers
SNPs: Single nucleotide polymorphisms
TKIs: Tyrosine kinase inhibitors
UHKT: Institute of Hematology and Blood Transfusion
