# Supplementary material for: Genotypes of SLC22A4 and SLC22A5 regulatory loci are predictive of the response of chronic myeloid leukemia patients to imatinib treatment
Source: J Exp Clin Cancer Res. 2017 Apr 18;36:55. doi: 10.1186/s13046-017-0523-3 (PMC5395939; doi:10.1186/s13046-017-0523-3)
Supplement: Supplementary file 1 — Characteristics of the patients. (DOCX 28 kb) [file 13046_2017_523_MOESM1_ESM.docx]

Supplementary Table S1. Characteristics of the patients.

|  | Cohort of patients for initial high-throughput screening | Patients added to the cohort |
| --- | --- | --- |
| Number | 83 | 46 |
| Median of age (range) | 54 (18-84) | 56 (19-80) |
| Sex | Female n=35  Male n=48 | Female n=19  Male n=27 |
| EUTOS score | Low n=64  High n=17  N/A n=2 | Low n=41  High n=5 |
| Sokal score | Low n=34  Intermediate n=23  High n=23  N/A n=3 | Low n=21  Intermediate n=18  High n=7 |
| Hasford score | Low n=27  Intermediate n=39  High n=14  N/A n=3 | Low n=14  Intermediate n=26  High n=6 |
| First line treatment | Imatinib 400 mg daily  Dose reduction in 21 patients (200-300 mg/day; median 1.9 months since imatinib start, range 0.2-7.4) | Imatinib 400 mg daily |
| Response to treatment at 12 months since IM start* | Optimal n=40  Warning n=13  Failure n=30 | Optimal n=32  Warning n=4  Failure n=10 |
| Stable MMR achievement**  Median months since imatinib start (range) | n=51  9.1 (3-51)  38 patients achieved MMR at 12 months | n=33  7.7 (4-21)  32 patients achieved MMR at 12 months |
| Therapy switch to dasatinib or nilotinib due to imatinib failure, response lost or progression (median months after imatinib treatment; range) | n=27  29.8 (12-113) | n=11  22.3 (12-62) |
| Death related to CML during imatinib treatment | n=3 | n=2 |
| Death un-related to CML during imatinib treatment | n=5 | n=2 |

* Response to imatinib treatment was evaluated according to the ELN recommendation^3^

** Excluding BCR-ABL1 levels fluctuation

Eighty three CML patients were diagnosed from 2004 to 2013. The minimum follow-up time of the patients on first-line imatinib treatment was 12 months after the initiation. The imatinib standard dose was reduced in 21 patients due to intolerance. The group of 46 added patients were diagnosed from 2004 to 2014 and were treated with imatinib as the first-line therapy at 400 mg/day. The minimum follow-up time of the patients on first-line imatinib treatment was 12 months after the initiation.
