# Supplementary material for: Genotypes of SLC22A4 and SLC22A5 regulatory loci are predictive of the response of chronic myeloid leukemia patients to imatinib treatment
Source: J Exp Clin Cancer Res. 2017 Apr 18;36:55. doi: 10.1186/s13046-017-0523-3 (PMC5395939; doi:10.1186/s13046-017-0523-3)

Supplementary Figure S1 A colormap of genotypes distribution among optimally and non-optimally responding patients to first-line imatinib treatment at 12 months. Each square illustrates each genotyped SNP for each patient. Red squares = minor allele homozygotes; pink squares = heterozygotes; white squares = major allele homozygotes; gray square = not analyzed.


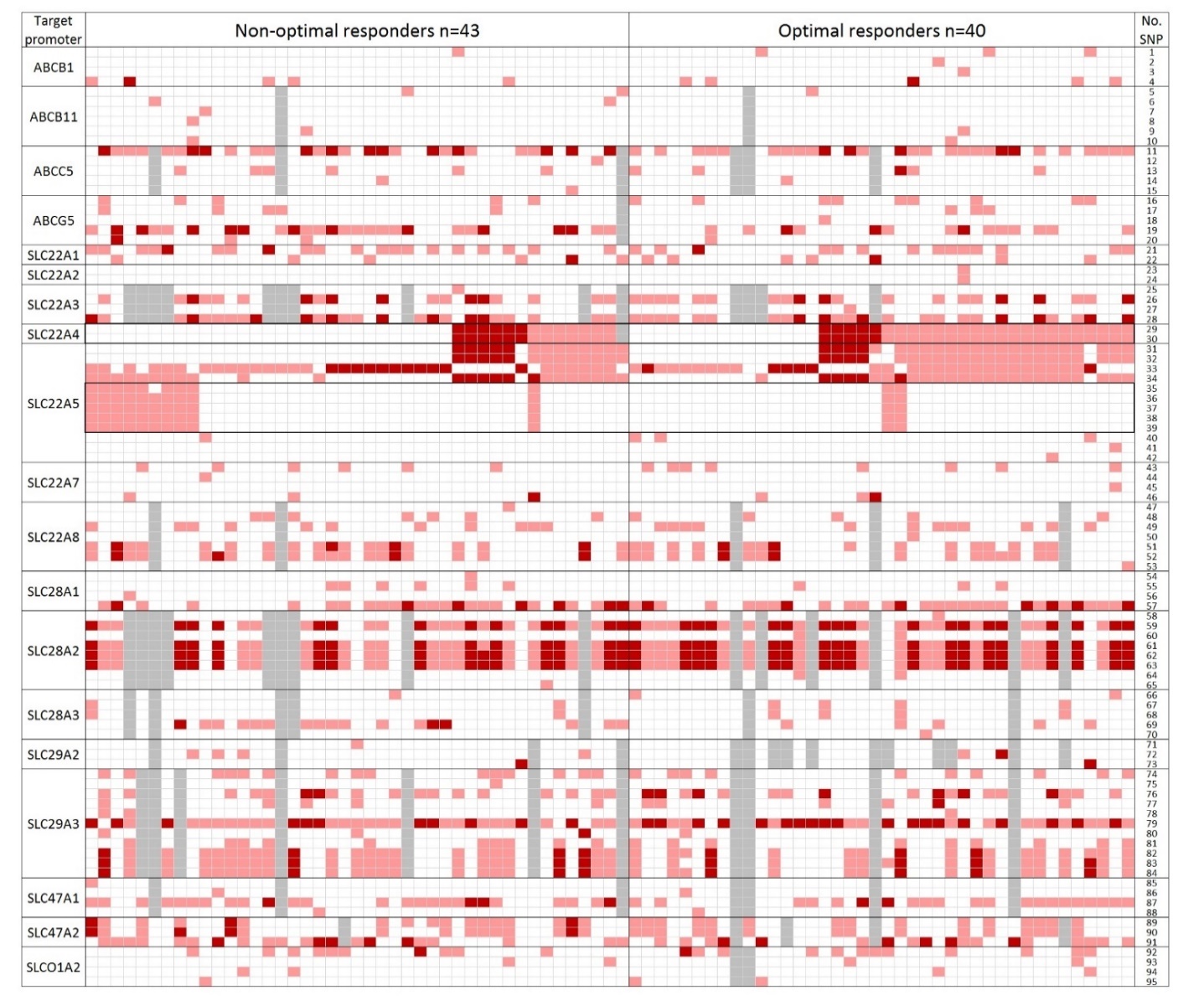


Supplementary Figure S2 Genotype frequencies of the rs460089 and rs460271 in patients with optimal and non-optimal response to imatinib at 12 months. 1 – Initial cohort of 83 patients; 2 – An independent group of added patients. Note – the graphs illustrate frequencies of genotypes of rs460089, which exactly reflect genotypes frequencies of rs460271.


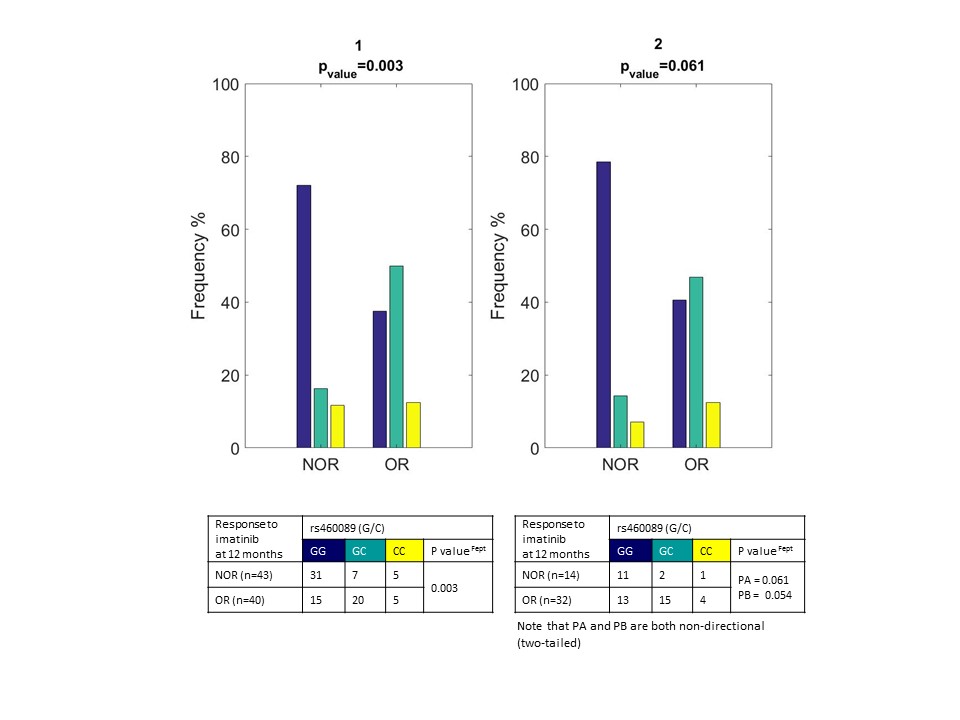


Legend: NOR - patients with non-optimal response to treatment; OR - patients with optimal treatment response.

Supplementary Figure S3 Genotype frequencies of a. rs13180043 (*SLC22A5*) and b. rs1050152 (*SLC22A4*, exon 9) in patients with optimal and non-optimal response to imatinib at 12 months. Note – the graph a. illustrates frequencies of genotypes of rs13180043, which exactly reflect genotypes frequencies of rs4646298, rs13180169, rs1310186, and rs13180295.


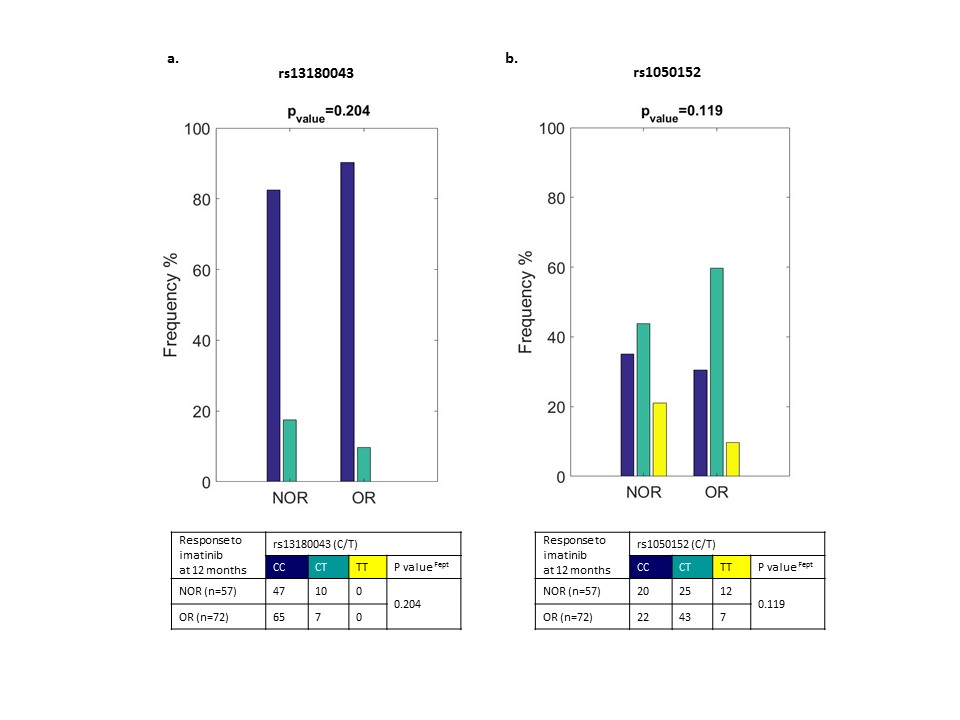


Legend: NOR - patients with non-optimal response to treatment; OR - patients with optimal treatment response.

Supplementary Figure S4 Relative mRNA levels of *SLC22A4* and *SLC22A5* in tested cell lines. a. Graph shows expression in all 8 cell lines. b. Graph shows expression of cell lines carrying rs460089-GG_rs2631365-TC or rs460089-GC_rs2631365-TC genotypes.


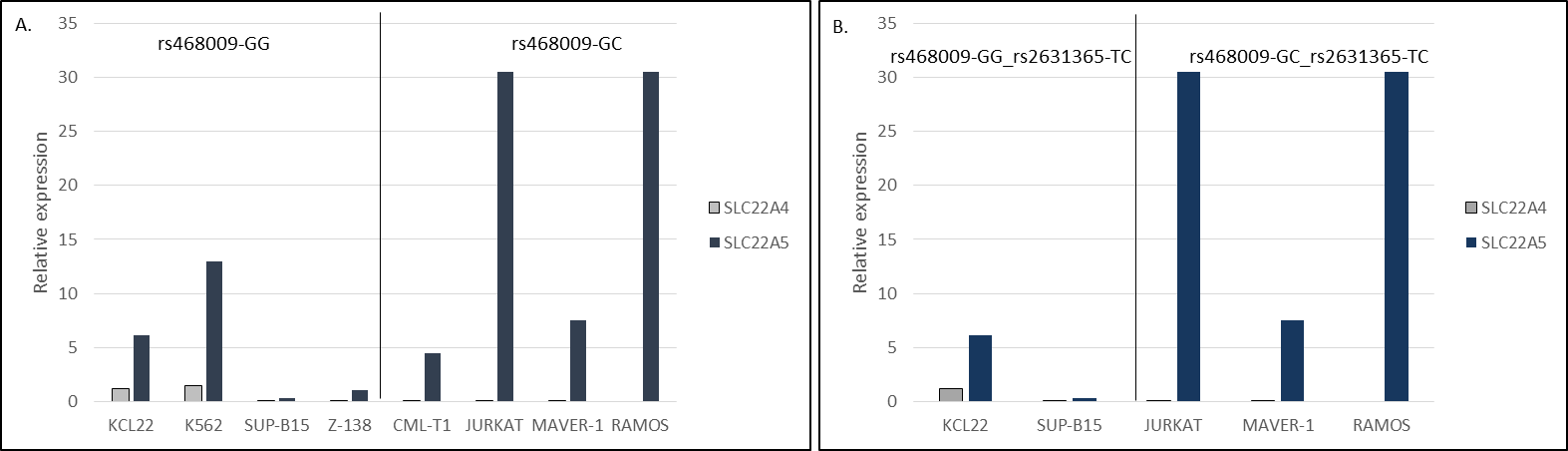

Supplement: Supplementary file 5 — A colormap of genotypes distribution among optimally and non-optimally responding patients to first-line imatinib treatment at 12 months. Each square illustrates each genotyped SNP for each patient. Red squares = minor allele homozygotes; pink squares = heterozygotes; white squares = major allele homozygotes; gray square = not analyzed. Figure S2. Genotype frequencies of the rs460089 and rs460271 in patients with optimal and non-optimal response to imatinib at 12 months. 1 – Initial cohort of 83 patients; 2 – An independent group of added patients. Note – the graphs illustrate frequencies of genotypes of rs460089, which exactly reflect genotypes frequencies of rs460271. Figure S3. Genotype frequencies of a. rs13180043 (SLC22A5) and b. rs1050152 (SLC22A4, exon 9) in patients with optimal and non-optimal response to imatinib at 12 months. Note – the graph a. illustrates frequencies of genotypes of rs13180043, which exactly reflect genotypes frequencies of rs4646298, rs13180169, rs1310186, and rs13180295. Figure S4. Relative mRNA levels of SLC22A4 and SLC22A5 in tested cell lines. a. Graph shows expression in all eight cell lines. b. Graph shows expression of cell lines carrying rs460089-GG_rs2631365-TC or rs460089-GC_rs2631365-TC genotypes. (DOCX 687 kb) [file 13046_2017_523_MOESM5_ESM.docx]
